# Supplementary material for: Locus of control is associated with tobacco and alcohol consumption in young adults of the Avon Longitudinal Study of Parents and Children
Source: R Soc Open Sci. 2019 Mar 27;6(3):181133. doi: 10.1098/rsos.181133 (PMC6458410; doi:10.1098/rsos.181133)
Supplement: 1. Association between the locus of control score at age 8 and smoking and drinking outcomes. 2. Supplementary Tables [file rsos181133supp1.docx]

**SUPPLEMENTARY MATERIAL**

**INTRODUCTION**

LoC changes across the lifespan; it tends to be more external in children and progressively becomes more internal in adolescents, when thinking becomes more abstract, hypothetical and critical. It then remains relatively stable in adolescence (1) and adulthood (2) with a trend to becoming yet more internal in later adulthood (3).

**METHODS**

LoC was assessed in participants selected from the ALSPAC birth cohort at approximately 8 years of age (mean 8.6 years, SD 0.3) by means of a brief version of the Nowiki-Stickland Internal-External scale consisting of 12 items (4) (see Supplementary Table 1).

**Statistical analysis**

The difference between LoC at 8 years and adolescents at 16 years was assessed with a Wilcoxon matched-pairs signed-rank test for matching pairs of measurements (including only individuals with a LoC score both at age 8 and at age 16; N = 3,196) and with a two-sample Mann-Whitney test to compare all subjects.

Associations between LoC at age 8 (measured as a continuous variable) and all outcomes (all binary) were assessed via logistic regressions. All analyses were conducted in Stata (version 14). Models adjusted for all confounders are reported as our main results. Models adjusted for sex and age analyses are reported in Supplementary Tables 6-7. Results for the complete case samples are reported in the main text.

**RESULTS**

**Characteristics of Participants**

For a detailed description of the study sample, including age, sex, IQ, maternal smoking and drinking, maternal education, paternal social class, smoking status, FTND and AUDIT scores, see Supplementary Tables 2. For associations between locus of control at 8 and potential confounders see Supplementary Table 3.

LoC data were available on 6,173 children. Children at age 8 had a neutral LoC (median = 6, IQR 4,7; mean = 5.98, SD 2.07) while adolescents at age 16 had a more internal LoC (median = 3, IQR 2,4; mean = 3.21, SD 2.12) (P < 0.001 for both the analysis including only matching measurement pairs - Wilcoxon matched-pairs signed-rank test- and the one including all subjects - two-sample Mann-Whitney test).

**Tobacco Use**

*LoC at age 8 and smoking status at 17 and 21 years*. LoC at age 8 was not associated with being at least a weekly smoker at age 17 (OR 0.95, 95% CI 0.89 to 1.02, P = 0.15) or at age 21 (OR 0.98, 95% CI 0.91 to 1.04, P = 0.47; Supplementary Table 6 and Supplementary Figure 1).

*LoC at age 8 and nicotine dependence at 17 and 21 years*. LoC at age 8 was not associated with nicotine dependence in daily smokers at age 17 (OR 0.88, 95% CI 0.72 to 1.07, P = 0.20) or 21 (OR 1.00, 95% CI 0.84 to 1.19, P = 1.00; Supplementary Table 6 and Supplementary Figure 1).

**Alcohol Use**

*LoC at age 8 and alcohol misuse at 17 and 21 years*. There was no evidence for an association of LoC at age 8 with the AUDIT score at 17 years (OR 0.99, 95% CI 0.94 to 1.03, P = 0.62) and 21 years (OR 0.99, 95% CI 0.94 to 1.05, P = 0.85; Supplementary Table 7 and Supplementary Figure 2).

**DISUCSSION AND CONCLUSION**

We found insufficient evidence for an association between LoC at age 8 and subsequent smoking status, nicotine dependence or drinking behaviour. In contrast, as reported in the main text, having a more external LoC at age 16, is associated with smoking behaviours at both age 17 and 21 and with hazardous use of alcohol at 17.

Interestingly, LoC at 8 is still developing and, therefore, it cannot be considered a stable measure especially compared to LoC at 16. Hence, it is not surprising to see these differences in our results according to the age at which LoC was assessed. In facts, we observed a more internal LoC in 16 year old adolescents compared to 8 year old children, consistent with previous reports (1).This shift towards internalization with age is likely due to fundamental changes occurring between age 8 and age 16 that involve cognitive development and changes in social demands and expectations. Our results suggest interventions may need to be delivered during certain critical periods, as only LoC at age 16 (and not at age 8) was associated with tobacco and alcohol use.

**Supplementary Table 1.** **Twelve-item brief version of the Nowiki-Stickland Internal External scale.**

1. Do you feel that wishing can make good things happen?
2. Are people nice to you no matter what you do?
3. Do you usually do badly in your schoolwork even when you try hard?
4. When a friend is angry with you is it hard to make that friend like you again?
5. Are you surprised when your teacher praises you for your work in school?
6. When bad things happen to you is it usually someone else's fault?
7. Is doing well in your schoolwork just a matter of "luck" for you?
8. Are you often blamed for things that just aren't your fault?
9. When you get into an argument or fight is it usually the other person's fault?
10. Do you think that preparing for things is a waste of time?
11. When nice things happen to you is it usually because of "luck"?
12. Does planning ahead make good things happen?

**Supplementary Table 2. Description of study sample with LoC score at age 16 and 8 and 17 and 21 year data.**

N = number of individuals that have taken the 8 year and the 16 year clinic and for which the measure is available. LoC = Locus of Control. IQ = intelligence quotient. CSE = certificate of secondary education; Vocational is an apprenticeship qualification. O level is more academic than the CSE and vocational. CSE, vocational and O level were qualifications taken at 16 years. A levels were examinations taken at 18 years. FTND = Fagerström Test of Nicotine Dependence. AUDIT = Alcohol Use Disorders Identification Test.

|  |  | **LoC at age 16** | | | | **LoC at age 8** | | | |
| --- | --- | --- | --- | --- | --- | --- | --- | --- | --- |
|  |  | **17 years** | | **21 years** | | **17 years** | | **21 years** | |
| **MEASURE** | **CATEGORY** | **N** | **MEAN (SD)** | **N** | **MEAN (SD)** | **N** | **MEAN (SD)** | **N** | **MEAN (SD)** |
| LoC score |  | 3258 | 3.09 (2.07) | 3046 | 3.07 (2.05) | 3550 | 5.84 (2.09) | 2832 | 5.75 (2.07) |
| IQ |  | 2897 | 111.81 (21.37) | 2053 | 112.49 (21. 78) | 3506 | 109.74 (21.76) | 2801 | 111.24 (21.04) |
|  |  |  | **N (%)** |  | **N (%)** |  | **N (%)** |  | **N (%)** |
| Sex | Male | 1330 | 40.82 | 1139 | 37.39 | 1613 | 45.44 | 1134 | 40.04 |
|  | Female | 1928 | 59.18 | 1907 | 62.61 | 1937 | 54.56 | 1698 | 59.96 |
| Maternal smoking  (at child age 12) | No | 2446 | 87.05 | 2252 | 86.48 | 2555 | 86.08 | 2071 | 86.18 |
|  | Yes | 364 | 12.95 | 352 | 13.52 | 413 | 13.92 | 332 | 13.82 |
| Maternal Drinking  (Total weekly units at child age 12) | 0 | 504 | 18.48 | 477 | 19.01 | 527 | 18.36 | 423 | 18.21 |
|  | 1 - 5 | 980 | 35.94 | 894 | 35.63 | 1023 | 35.64 | 828 | 35.64 |
|  | 6 - 10 | 669 | 24.53 | 615 | 24.51 | 726 | 25.30 | 594 | 25.57 |
|  | 11 – 15 | 303 | 11.11 | 276 | 11.00 | 320 | 11.15 | 258 | 11.11 |
|  | 16+ | 271 | 9.94 | 247 | 9.84 | 274 | 9.55 | 220 | 9.47 |
| Maternal education | CSE | 284 | 9.31 | 287 | 10.03 | 327 | 9.77 | 241 | 9.01 |
|  | Vocational | 193 | 6.33 | 183 | 6.40 | 246 | 7.35 | 197 | 7.36 |
|  | O level | 995 | 32.62 | 932 | 32.58 | 1139 | 34.04 | 871 | 32.56 |
|  | A level | 900 | 29.51 | 816 | 28.52 | 973 | 29.08 | 793 | 29.64 |
|  | Degree | 678 | 22.23 | 643 | 22.47 | 661 | 19.75 | 573 | 21.42 |
| Paternal occupation | Professional occupations | 474 | 16.64 | 456 | 16.97 | 476 | 15.30 | 407 | 16.22 |
|  | Managerial and technical occupations | 1114 | 39.10 | 1061 | 39.49 | 1177 | 37.83 | 973 | 38.78 |
|  | Non manual skilled occupations | 354 | 12.43 | 334 | 12.43 | 389 | 12.50 | 327 | 13.03 |
|  | Manual skilled occupations | 680 | 23.87 | 631 | 23.48 | 791 | 25.43 | 592 | 23.60 |
|  | Partly skilled occupations | 184 | 6.46 | 166 | 6.18 | 221 | 7.10 | 168 | 6.70 |
|  | Unskilled occupations | 43 | 1.51 | 39 | 1.45 | 57 | 1.83 | 42 | 1.67 |
| Smoking status | Not a weekly smoker | 2371 | 85.20 | 2473 | 82.74 | 2456 | 83.25 | 2242 | 80.97 |
|  | At least a weekly smoker | 412 | 14.80 | 516 | 17.26 | 494 | 16.75 | 527 | 19.03 |
| FTND | Score < 4 | 197 | 68.17 | 253 | 72.49 | 244 | 67.03 | 263 | 71.47 |
|  | Score ≥ 4 | 92 | 31.83 | 96 | 27.51 | 120 | 32.97 | 105 | 28.53 |
| AUDIT | Score < 8 | 1733 | 62.74 | 1303 | 45.66 | 1733 | 59.43 | 1165 | 44.13 |
|  | Score ≥ 8 | 1029 | 37.26 | 1551 | 54.34 | 1183 | 40.57 | 1475 | 55.87 |

**Supplementary Table 3. Associations between locus of control at 8 and 16 years and potential confounders.**

Beta represents change in LoC score from linear regression. LoC = Locus of Control. IQ = intelligence quotient. CSE = certificate of secondary education; Vocational is an apprenticeship qualification. O level is more academic than the CSE and vocational. CSE, vocational and O level were qualifications taken at 16 years. A levels were examinations taken at 18 years.

|  | | **LoC AT 16** | | | **LoC AT 8** | | |
| --- | --- | --- | --- | --- | --- | --- | --- |
| **MEASURE** | **CATEGORY** | **N** | **BETA (95% CI)** | **P-value** | **N** | **BETA (95% CI)** | **P-value** |
| Sex | Males | 4,656 | - | - | 6,173 | - | - |
|  | Females |  | 0.39 (0.26, 0.51) | <0.001 |  | 0.11 (0.00, 0.21) | 0.041 |
| IQ |  | 3,691 | -.02 (-0.02, -0.02) | <0.001 | 6,102 | -0.03 (-0.03, -0.02) | <0.001 |
| Maternal smoking | No | 3,840 | - | - | 4,542 | - | - |
|  | Yes |  | 0.42 (0.23, 0.60) | <0.001 |  | 0.34 (0.17, 0.50) | <0.001 |
| Maternal drinking | 0 | 3,702 | - | - | 4,375 | - | - |
|  | 1 - 5 |  | -0.14 (-0.33, 0.05) | 0.140 |  | -0.18 (-0.35, -0.00) | 0.049 |
|  | 6 - 10 |  | -0.24 (-0.45, -0.03) | 0.022 |  | -0.26 (-0.45, -0.00) | 0.007 |
|  | 11 – 15 |  | -0.48 (-0.74, -0.22) | <0.001 |  | -0.40 (-0.64, -0.16) | 0.001 |
|  | 16+ |  | -0.31 (-0.57, -0.05) | 0.020 |  | -0.33 (-0.57 - -0.90) | 0.007 |
| Maternal education | CSE | 4,329 | - | - | 5,709 | - | - |
|  | Vocational |  | -0.12 (-0.41, 0.16) | 0.399 |  | -0.17 (-0.40, 0.05) | 0.13 |
|  | O level |  | -0.52 (-0.73, -0.31) | <0.001 |  | -0.39 (-0.56, -0.22) | <0.001 |
|  | A level |  | -0.87 (-1.09, -0.66) | <0.001 |  | -0.89 (-1.07, -0.71) | <0.001 |
|  | Degree |  | -1.33 (-1.56, -1.10) | <0.001 |  | -1.41 (-1.60, -1.21) | <0.001 |
| Paternal occupation | Professional occupations | 4,011 | - | - | 5,251 | - | - |
|  | Managerial/technical occupations |  | 0.29 (0.10, 0.48) | 0.002 |  | 0.31 (0.13, 0.49) | 0.001 |
|  | Non manual skilled occupations |  | 0.56 (0.31, 0.80) | <0.001 |  | 0.31 (0.27, 0.71) | <0.001 |
|  | Manual skilled occupations |  | 0.86 (0.66, 1.07) | <0.001 |  | 0.91 (0.72, 1.10) | <0.001 |
|  | Partly skilled occupations |  | 1.05 (0.76, 1.34) | <0.001 |  | 0.95 (0.71, 1.20) | <0.001 |
|  | Unskilled occupations |  | 1.45 (0.93, 1.98) | <0.001 |  | 1.18 (0.79, 1.58) | <0.001 |

|  | | **WEEKLY SMOKING AT AGE 17** | | | **WEEKLY SMOKING AT AGE 21** | | | **FTND AT AGE 17** | | | **FTND AT AGE 21** | | |
| --- | --- | --- | --- | --- | --- | --- | --- | --- | --- | --- | --- | --- | --- |
| **MEASURE** | **CATEGORY** | **N** | **OR (95% CI)** | **P-value** | **N** | **OR (95% CI)** | **P-value** | **N** | **OR (95% CI)** | **P-value** | **N** | **OR (95% CI)** | **P-value** |
| Sex | Males | 4,090 | - | - | 4,128 | - | - | 510 | - | - | 562 | - | - |
|  | Females |  | 1.14 (0.97, 1.34) | 0.123 |  | 1.13 (0.96, 1.33) | 0.132 |  | 1.19 (0.82, 0.73) | 0.366 |  | 0.85 (0.57, 1.28) | 0.436 |
| IQ | Per additional point | 3.435 | 0.99 (0.99, 0.99) | <0.001 | 3,209 | 0.99 (0.99, 1.00) | 0.007 |  | 0.98 (0.97, 0.99) | <0.001 | 420 | 0.98 (0.97, 0.99) | 0.002 |
| Maternal smoking | No | 3,206 | - | - | 3,237 | - | - | 376 | - | - | 403 | - | - |
|  | Yes |  | 2.84 (2.26, 3.57) | <0.001 |  | 2.70 (2.17, 3.35) | <0.001 |  | 1.43 (0.90, 2.27 | 0.130 |  | 1.49 (0.86, 2.28) | 0.179 |
| Maternal drinking | 0 | 3,083 | - | - | 3,123 | - | - | 365 | - | - | 388 | - | - |
|  | 1 – 5 |  | 1.02 (0.77, 1.35) | 0.875 |  | 1.09 (0.83, 1.42) | 0.547 |  | 1.17 (0.63, 2.15) | 0.602 |  | 1.39 (0.71, 2.76) | 0.337 |
|  | 6 – 10 |  | 1.00 (0.73, 1.34) | 0.875 |  | 1.21 (0.91, 1.61) | 0.178 |  | 0.98 (0.49, 1.97) | 0.955 |  | 1.02 (0.51, 2.05) | 0.951 |
|  | 11 – 15 |  | 1.32 (0.93, 1.87) | 0.115 |  | 1.32 (0.94, 1.86) | 0.112 |  | 0.77 (0.34, 1.72) | 0.523 |  | 0.49 (0.22, 1.10) | 0.083 |
|  | 16+ |  | 1.75 (1.22, 2.51) | 0.002 |  | 1.88 (1.34, 2.63) | <0.001 |  | 0.94 (0.39, 2.23) | 0.525 |  | 0.55 (0.26, 1.19) | 0.128 |
| Maternal education | CSE | 3,724 | - | - | 3,842 | - | - | 457 | - | - | 508 | - | - |
|  | Vocational |  | 0.76 (0.49, 1.11) | 0.157 |  | 0.65 (0.45, 0.93) | 0.019 |  | 1.87 (0.87, 4.02) | 0.106 |  | 0.60 (0.20, 1.83) | 0.372 |
|  | O level |  | 0.67 (0.51, 0.88) | 0.004 |  | 0.61 (0.48, 0.79) | <0.001 |  | 1.37 (0.78, 2.39) | 0.267 |  | 0.33 (0.15, 0.71) | 0.005 |
|  | A level |  | 0.58 (0.44, 0.77) | <0.001 |  | 0.61 (0.47, 0.80) | <0.001 |  | 0.75 (0.40, 1.41) | 0.368 |  | 0.23 (0.11, 0.50) | <0.001 |
|  | Degree |  | 0.51 (0.37, 0.69) | <0.001 |  | 0.49 (0.37, 0.66) | <0.001 |  | 0.78 (0.36, 1.69) | 0.525 |  | 0.24 (020, 1.83) | 0.001 |
| Paternal occupation | Professional occupations | 3,457 | - | - | 3,567 | - | - | 408 | - | - | 448 | - | - |
|  | Managerial and  technical occupations |  | 1.51 (1.13, 2.09) | 0.007 |  | 1.45 (1.11, 1.91) | 0.007 |  | 1.70 (0.60, 4.78) | 0.315 |  | 1.37 (0.66, 2.84) | 0.393 |
|  | Non manual skilled occupations |  | 1.50 (1.03, 2.19) | 0.036 |  | 1.20 (0.85, 1.70) | 0.299 |  | 3.12 (1.02, 9.58) | 0.047 |  | 1.36 (0.55, 3.35) | 0.505 |
|  | Manual skilled occupations |  | 1.89 (1.38, 2.60) | <0.001 |  | 1.47 (1.10, 1.97) | 0.009 |  | 2.75 (0.99, 7.62) | 0.052 |  | 1.85 (0.85, 4.02) | 0.118 |
|  | Partly skilled occupations |  | 2.61 (1.38, 3.10) | 0.001 |  | 1.63 (1.11, 2.40) | 0.012 |  | 2.51 (0.79, 7.93) | 0.117 |  | 2.64 (0.93, 7.51) | 0.069 |
|  | Unskilled occupations |  | 2.71 (1.51, 4.87) | 0.001 |  | 1.70 (0.90, 3.22) | 0.103 |  | 6.68 (1.69, 26.45) | 0.007 |  | 6.72 (0.79, 57.25) | 0.081 |

**Supplementary Table 4. Associations between smoking status and potential confounders.**

Odds ratio for a) at least weekly smoking compared to less than weekly smoking; b) nicotine dependent smokers (FTND ≥ 4) compared to non-dependent smokers (FTND <4). FTND = Fagerström Test of Nicotine Dependence. LoC = Locus of Control. IQ = intelligence quotient at 8 years. CSE = certificate of secondary education; Vocational is an apprenticeship qualification. O level is more academic than the CSE and vocational. CSE, vocational and O level were qualifications taken at 16 years. A levels were examinations taken at 18 years.

**Supplementary Table 5. Associations between drinking measures and potential confounders.**

Odds ratio for individuals that make a hazardous use of alcohol (AUDIT > 8) compared to those who don’t (AUDIT ≤ 8). AUDIT = Alcohol Use Disorders Identification Test.

LoC = Locus of Control. IQ = intelligence quotient at 8 years. CSE = certificate of secondary education; Vocational is an apprenticeship qualification. O level is more academic than the CSE and vocational. CSE, vocational and O level were qualifications taken at 16 years. A levels were examinations taken at 18 year.

|  | | **AUDIT AT AGE 17** | | | **AUDIT AT AGE 21** | | |
| --- | --- | --- | --- | --- | --- | --- | --- |
| **MEASURE** | **CATEGORY** | **N** | **OR (95% CI)** | **P-value** | **N** | **OR (95% CI)** | **P-value** |
| **Sex** | Males | 4.041 | - | - | 3,943 | - | - |
|  | Females |  | 1.09 (0.85, 1.38) | 0.493 |  | 0.73 (0.54, 1.00) | 0.050 |
| **IQ** | Per additional point | 3,392 | 1.00 (0.99, 1.01) | 0.199 | 3,063 | 1.01 (1.01, 1.02) | 0.001 |
| **Maternal smoking** | No | 3,173 | - | - | 3,093 | - | - |
|  | Yes |  | 1.37 (0.88, 2.13) | 0.166 |  | 0.91 (0.57, 1.46) | 0.708 |
| **Maternal drinking** | 0 | 3,050 | - | - | 2,985 | - | - |
|  | 1 - 5 |  | 1.98 (1.39, 2.82) | <0.001 |  | 2.06 (1.35, 3.14) | 0.001 |
|  | 6 - 10 |  | 2.66 (1.75, 4.05) | <0.001 |  | 3.39 (1.97, 5.83) | <0.001 |
|  | 11 – 15 |  | 2.15 (1.28, 3.61) | 0.004 |  | 2.83 (1.41, 5.67) | 0.003 |
|  | 16+ |  | 3.42 (1.73, 6.75) | <0.001 |  | 3.04 (1.42, 6.52) | 0.004 |
| **Maternal education** | CSE | 3,682 | - | - | 3,671 | - | - |
|  | Vocational |  | 1.95 (0.68, 2.08) | 0.531 |  | 2.20 (1.15, 4,19) | 0.016 |
|  | O level |  | 1.45 (0.97, 2.17) | 0.069 |  | 2.34 (1.53, 3.59) | <0.001 |
|  | A level |  | 1.63 (1.10, 2.48) | 0.024 |  | 2.35 (1.51, 3.66) | <0.001 |
|  | Degree |  | 1.31 (0.85, 2.03) | 0.221 |  | 2.79 (1.69, 4.62) | <0.001 |
| **Paternal occupation** | Professional occupations | 3,417 | - | - | 3,409 | - | - |
|  | Managerial and  technical occupations |  | 1.10 (0.74, 1.64) | 0.635 |  | 1.15 (0.69, 1.93) | 0.589 |
|  | Non manual skilled occupations |  | 1.13 (0.68, 1.90) | 0.629 |  | 0.86 (0.46, 1.59) | 0.623 |
|  | Manual skilled occupations |  | 0.99 (0.65, 1.90) | 0.979 |  | 0.69 (0.41, 1.16) | 0.167 |
|  | Partly skilled occupations |  | 1.01(0.56, 1.81) | 0.973 |  | 0.73 (0.36, 1.48) | 0.382 |
|  | Unskilled occupations |  | 0.76 (0.32, 1.76) | 0.514 |  | 0.81 (0.24, 2.80) | 0.743 |

**Supplementary Table 6****.** **Association between locus of control at 16 and at 8 years and smoking status and nicotine dependence at 17 and 21 years.**

LoC = Locus of Control. Smoking status was defined as being at least a weekly smoker vs. a less than weekly smoker. FTND = Fagerström Test for Nicotine Dependence. Regressions were adjusted for: 1. age, sex; 2. age, sex, IQ at age 8, maternal smoking at 12, maternal drinking at 12, maternal education and paternal occupation.

|  | **SMOKING STATUS AT 17** | | | **SMOKING STATUS AT 21** | | | **FTND at 17** | | | **FTND at 21** | | |
| --- | --- | --- | --- | --- | --- | --- | --- | --- | --- | --- | --- | --- |
| **LoC AT 16** | **N** | **OR (95% CI)** | **P-value** | **N** | **OR (95% CI)** | **P-value** | **N** | **OR (95% CI)** | **P-value** | **N** | **OR (95% CI)** | **P-value** |
| **Model 1** | 2,783 | 1.20 (1.14, 1.26) | <0.001 | 2,989 | 1.16 (1.11, 1.22) | <0.001 | 289 | 1.16 (1.04, 1.29) | 0.006 | 349 | 1.19 (1.07, 1.32) | 0.013 |
| **Model 2** | 1,922 | 1.18 (1.10, 1.25) | <0.001 | 1,899 | 1.14 (1.07, 1.21) | <0.001 | 181 | 1.26 (1.05, 1.51) | 0.013 | 194 | 1.25 (1.05, 1.49) | 0.001 |
| **LoC AT 8** | **N** | **OR (95% CI)** | **P-value** | **N** | **OR (95% CI)** | **P-value** | **N** | **OR (95% CI)** | **P-value** | **N** | **OR (95% CI)** | **P-value** |
| **Model 1** | 2,272 | 1.01 (0.95, 1.06) | 0.829 | 2,278 | 1.02 (0.97, 1.07) | 0.494 | 233 | 1.02 (0.90, 1.17) | 0.687 | 368 | 1.05 (0.94, 1.18) | 0.394 |
| **Model 2** | 1,732 | 0.95 (0.89, 1.02) | 0.148 | 1,715 | 0.98 (0.91, 1.04) | 0.467 | 165 | 0.88 (0.72, 1.07) | 0.199 | 219 | 1.00 (0.84, 1.19) | 0.999 |

**Supplementary Table 7. Association between locus of control at 16 and 8 years** **and alcohol consumption at 17 and 21 years.**

|  | **AUDIT at 17** | | | **AUDIT at 21** | | |
| --- | --- | --- | --- | --- | --- | --- |
| **LoC at 16** | **N** | **OR (95% CI)** | **P-value** | **N** | **OR (95% CI)** | **P-value** |
| **Model 1** | 2,762 | 1.09 (1.05, 1.13) | <0.001 | 2,854 | 0.97 (0.93, 1.00) | 0.079 |
| **Model 2** | 1,905 | 1.09 (1.04, 1.15) | <0.001 | 1,816 | 1.01 (0.96, 1.06) | 0.684 |
| **LoC at 8** | **N** | **OR (95% CI)** | **P-value** | **N** | **OR (95% CI)** | **P-value** |
| **Model 1** | 2,916 | 0.97 (0.93, 1.00) | 0.080 | 2,173 | 0.94 (0.90, 0.98) | 0.004 |
| **Model 2** | 2,087 | 0.99 (0.94, 1.03) | 0.615 | 1,640 | 0.99 (0.94, 1.05) | 0.851 |

LoC = Locus of Control. AUDIT = Alcohol Use Disorders Identification Test. Alcohol consumption was assessed via the AUDIT at 17 and 21 years. Regressions were adjusted for: 1. age, sex; 2. age, sex, IQ at age 8, maternal smoking at 12, maternal drinking at 12, maternal education and paternal occupation.

**Supplementary Table 8.** **Association between locus of control at 16 and smoking status and nicotine dependence at 17 and 21 years, including imputed data.**

|  | **SMOKING STATUS AT 17** | | | **SMOKING STATUS AT 21** | | | **FTND at 17** | | | **FTND at 21** | | |
| --- | --- | --- | --- | --- | --- | --- | --- | --- | --- | --- | --- | --- |
| **LoC AT 16** | **N** | **OR (95% CI)** | **P-value** | **N** | **OR (95% CI)** | **P-value** | **N** | **OR (95% CI)** | **P-value** | **N** | **OR (95% CI)** | **P-value** |
| **Model 1** | 4,090 | 1.21 (1.16, 1.27) | <0.001 | 4,951 | 1.18 (1.13, 1.23) | <0.001 | 510 | 1.14 (1.02, 1.27) | 0.024 | 562 | 1.18 (1.07, 1.30) | 0.001 |
| **Model 2** | 4,090 | 1.19 (1.13, 1.25) | <0.001 | 4,951 | 1.17 (1.11, 1.22) | <0.001 | 510 | 1.09 (0.96, 1.24) | 0.173 | 562 | 1.19 (1.06, 1.34) | 0.004 |

LoC = Locus of Control. Smoking status was defined as being at least a weekly smoker vs. a less than weekly smoker. FTND = Fagerström Test for Nicotine Dependence.Nicotine dependence was assessed via the FTND. Regressions were adjusted for: 1. age, sex; 2. age, sex, IQ, maternal smoking at 12, maternal drinking at 12, maternal education and paternal occupation.

**Supplementary Table 9. Association between locus of control at 16 years and alcohol consumption at 17 and 21 years, including imputed data.**

LoC = Locus of Control. AUDIT = Alcohol Use Disorders Identification Test. Alcohol consumption was assessed via the AUDIT at 17and 21 years.

Regressions were adjusted for: 1. age, sex; 2. age, sex, IQ, maternal smoking at 12, maternal drinking at 12, maternal education and paternal occupation.

|  | **AUDIT at 17** | | | **AUDIT at 21** | | |
| --- | --- | --- | --- | --- | --- | --- |
| **LoC at 16** | **N** | **OR (95% CI)** | **P-value** | **N** | **OR (95% CI)** | **P-value** |
| **Model 1** | 4,041 | 1.09 (1.05, 1.13) | <0.001 | 3,943 | 0.97 (0.93, 1.00) | 0.059 |
| **Model 2** | 4,041 | 1.11 (1.07, 1.15) | <0.001 | 3,943 | 1.01 (0.98, 1.05) | 0.447 |

**Supplementary figures**

**Figure 1. Association between locus of control at 8 and tobacco consumption at 17 and 21 years.** LoC = Locus of Control. FTND = Fagerström Test for Nicotine Dependence. **Dots** represent the odds ratio for being at least weekly smoking compared to less than weekly smoking and for being dependent on nicotine (FTND≥4) compared to not being dependent on nicotine (FTND<4). **Horizontal lines** represent 95% CIs. Regressions were adjusted for age, sex, IQ, maternal smoking at 12, maternal drinking at 12, maternal education and paternal occupation.

**
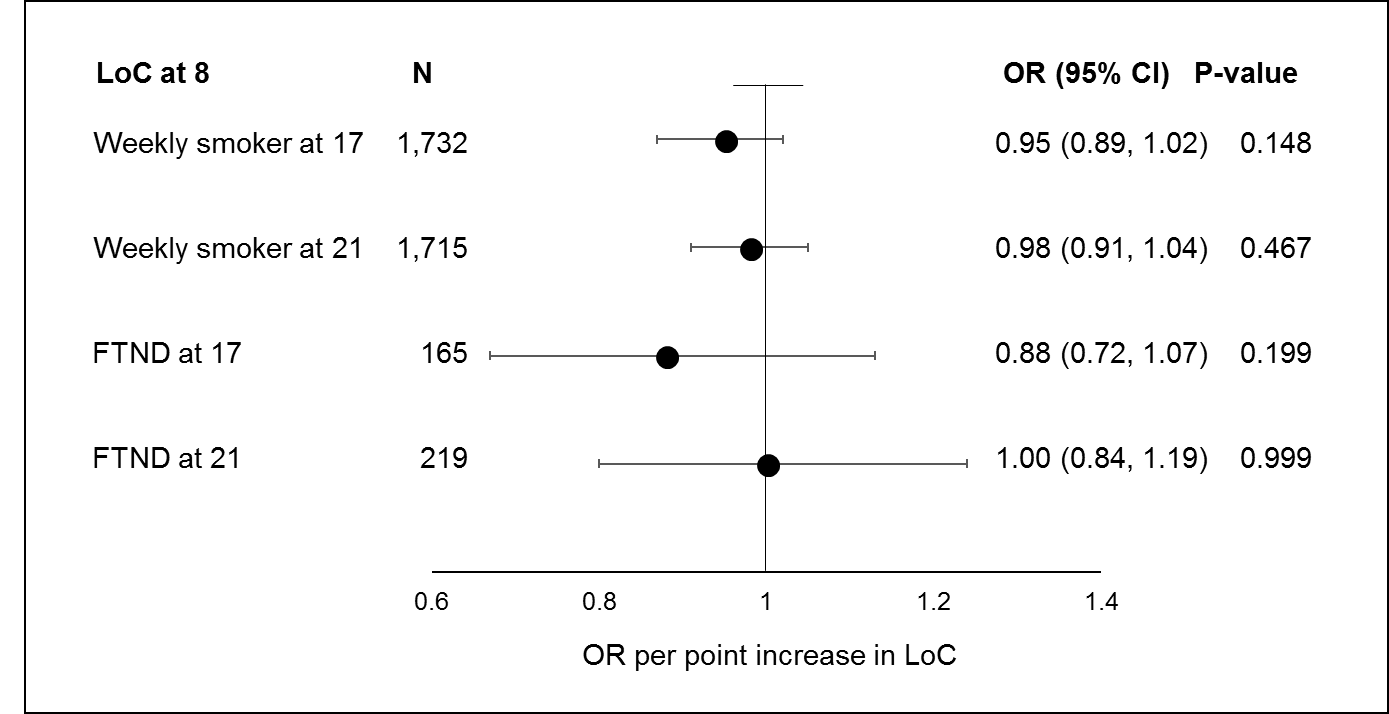
**

**Supplementary figure 2. Association between locus of control at 8 years and alcohol consumption at, 17 and 21 years.**

LoC = Locus of Control. AUDIT = Alcohol Use Disorders Identification Test. **Dots** represent the odds ratio for hazardous use of alcohol (AUDIT > 8) compared to non-hazardous (AUDIT ≤ 8) at 17 and 21 years. **Horizontal lines**represent 95% CIs. Regressions were adjusted for age, sex, IQ, maternal smoking at 12, maternal drinking at 12, maternal education and paternal occupation.

**
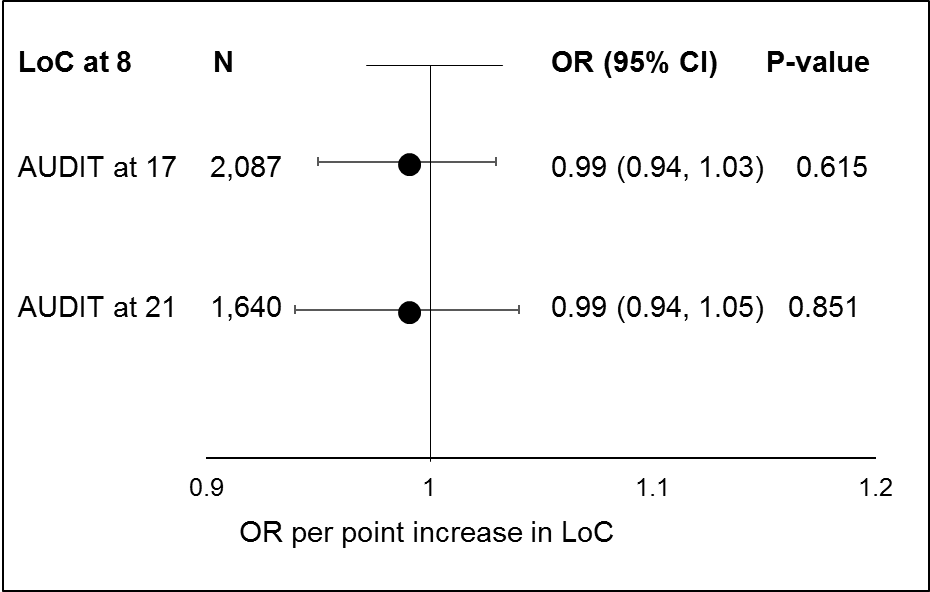
**

**ADDITIONAL ANALYSES**

**METHODS**

LoC was assessed in participants selected from the ALSPAC birth cohort (see main text). We have tested the association between LoC and Adverse Childhood Experiences (ACE) using a cumulative measure derived for 0-16 years, see https://wellcomeopenresearch.org/articles/3-106/v1, as well as between LoC and conduct disorder reported by the mother using the Strengths and Difficulties Questionnaire (SDQ) (5) at age 13. We then tested the association between LoC at 16 and tobacco and alcohol use adjusting for age, sex, IQ at age 8, maternal smoking at 12, maternal drinking at 12, maternal education, paternal occupation, ACE and conduct disorder.

**Statistical analysis**

Associations between LoC at age 16 and adverse childhood experiences, as well as between LoC at age 16 and conduct disorder, were assessed via linear regressions. To test the associations between LoC at 16 and tobacco and alcohol use we used logistic regressions adjusted for all confounders. All analyses were conducted in Stata (version 14).

**RESULTS**

Higher scores for ACE and conduct disorder were associated with a more external LoC (see Supplementary Table 10).

**Tobacco Use**

*LoC at 16 and smoking status at 17 and 21 years*. There was some evidence that a more external LoC at age 16 was associated with being at least a weekly smoker at age 17 (OR 1.13, 95% CI 1.02, 1.25, P = 0.02) and age 21 (OR 1.10, 95% CI 1.00, 1.22, P = 0.05; Supplementary Table 11).

*LoC at age 16 and nicotine dependence at 17 and 21 years.* Having a more external LoC at age 16 was associated with nicotine dependence at age 17 (OR 2.03, 95% CI 1.19 to 3.48, P = 0.01), and age 21 (OR 1.28, 95% CI 0.84 to 1.95, P = 0.02; Supplementary Table 11).

**Alcohol Use**

*LoC at age 16 and alcohol misuse at 17 and 21 years*. A more external LoC at age 16 was associated with higher odds of hazardous drinking on the AUDIT score at age 17 (OR 1.08, 95% CI 1.01 to 1.16, P < 0.03) but not at age 21 (OR 1.00, 95% CI 0.92 to 1.08, P = 0.91; Supplementary Table 11).

**DISUCSSION AND CONCLUSION**

We have tested the association between LoC at 16 and smoking and drinking outcomes adjusting for age, sex, IQ, maternal smoking, maternal drinking, maternal education, paternal occupation, adverse childhood experiences and conduct disorder. We obtained consistent results with the results reported in the main text, that is a more external LoC at age 16 was associated with higher odds of being at least a weekly smoker and being nicotine dependent at age 17 and age 21 and with greater odds of hazardous alcohol use at age 17 but not at age 21, but with a loss of power due to the very small sample size.

*Supplementary Table 10. Associations between locus of control at 16 years and adverse childhood experiences and conduct disorder.*

| **LoC AT 16** | | | |
| --- | --- | --- | --- |
| **MEASURE** | **N** | **BETA (95% CI)** | **P-value** |
| Adverse Childhood Experiences | 2,303 | 0.28 (0.21 – 0.36) | <0.001 |
| Conduct disorder | 2,539 | 0.21 (0.16 – 0.27) | <0.001 |

Beta represents change in LoC score from linear regression. LoC = Locus of Control. Higher scores for Adverse Childhood Experiences and conduct disorder are associated with a more external LoC.

*Supplementary Table 11. Association between locus of control at 16 and smoking status, nicotine dependence and alcohol consumption at 17 and 21 years.*

| **LoC AT 16** | | | |
| --- | --- | --- | --- |
| **MEASURE** | **N** | **OR (95% CI)** | **P-value** |
| Smoking status at 17 | 832 | 1.13 (1.02 – 1.25) | 0.02 |
| Smoking status at 21 | 819 | 1.10 (1.00 – 1.22) | 0.05 |
| FTND at 17 | 69 | 2.03 (1.19 – 3.48) | 0.01 |
| FTND at 21 | 78 | 1.28 (0.84 – 1.95) | 0.02 |
| AUDIT at 17 | 1,105 | 1.08 (1.01 – 1.16) | 0.03 |
| AUDIT at 21 | 1,105 | 1.00 (0.92 – 1.08) | 0.91 |

LoC = Locus of Control. Smoking status was defined as being at least a weekly smoker vs. a less than weekly smoker. FTND = Fagerström Test for Nicotine Dependence. AUDIT = Alcohol Use Disorders Identification Test. Regressions were adjusted for age, sex, IQ at age 8, maternal smoking at 12, maternal drinking at 12, maternal education, paternal occupation, Adverse Childhood Experiences and conduct disorder.

**REFERENCES**

1. Kulas H (1996). Locus of control in adolescence: a longitudinal study. Adolescence 31:721-729.
2. Wight R. G., Aneshensel C. S., Seeman M. E. (2003). Late lie cognition among men: a life course perspective on psychosocial experience. Arch Gerontol Geriatr 37:173-193
3. Lao R. (1976) Is internal-external control an age-related variable? J Psychol 92: 3-7.
4. Nowicki, S., & Strickland, B. R. (1973). A locus of control scale for children. Journal of Consulting and Clinical Psychology, 40*(*1), 148-154.
5. Goodman R (1997). The Strengths and Difficulties Questionnaire: a research note. J Child Psychol Psychiatry 38: 581–586.
